# Supplementary material for: Predicting visual function by interpreting a neuronal wiring diagram
Source: Nature. 2024 Oct 2;634(8032):113–23. doi: 10.1038/s41586-024-07953-5 (PMC11446822; doi:10.1038/s41586-024-07953-5)
Supplement: Supplementary file 6 — CRF and ERF predictions for individual TmY4 and TmY9 cells. Analogous to Supplementary Data 3, but for TmY target types. Shown are the top four monosynaptic pathways, the strongest pathway passing through each of the top ten intermediary types (ranking from Extended Data Fig. 7), and the trisynaptic pathway Tm1–TmY–Dm3–TmY (see the section entitled Prediction of spatial normalization). [file 41586_2024_7953_MOESM6_ESM.zip › DataS4/TmY9qΓèÑ/720575940622748381.pdf]

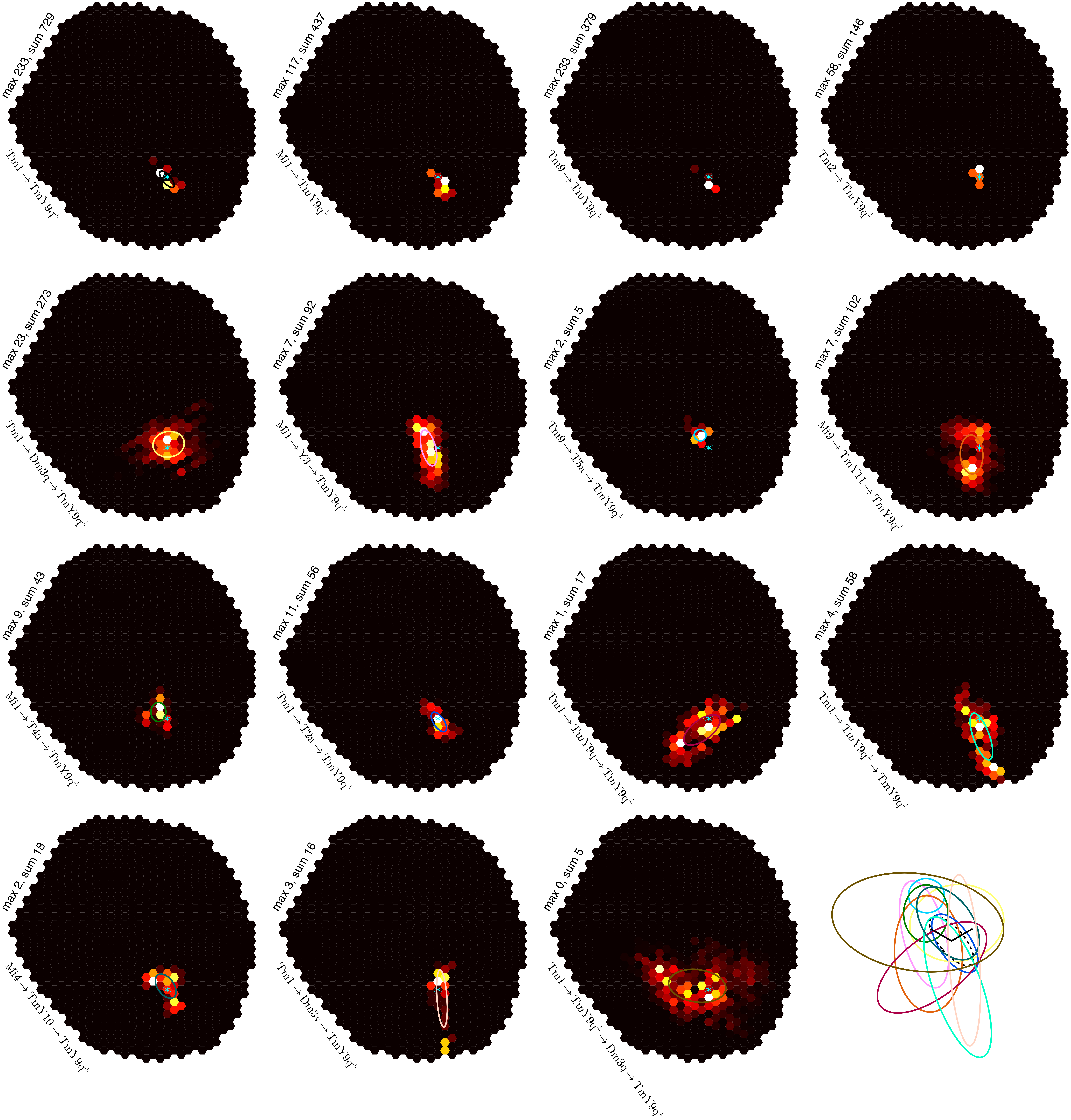

max 233, sum 729
Tm1 → TmY9q⊥
max 117, sum 437
Mi1 → TmY9q⊥
max 233, sum 379
Tm9 → TmY9q⊥
max 58, sum 146
Tm2 → TmY9q⊥
max 23, sum 273
Tm1 → Dm3q → TmY9q⊥
max 7, sum 92
Mi1 → Y3 → TmY9q⊥
max 2, sum 5
Tm9 → T5a → TmY9q⊥
max 7, sum 102
Mi9 → TmY11 → TmY9q⊥
max 9, sum 43
Mi1 → T4a → TmY9q⊥
max 11, sum 56
Tm1 → T2a → TmY9q⊥
max 1, sum 17
Tm1 → TmY9q → TmY9q⊥
max 4, sum 58
Tm1 → TmY9q⊥ → TmY9q⊥
max 2, sum 18
Mi4 → TmY10 → TmY9q⊥
max 3, sum 16
Tm1 → Dm3v → TmY9q⊥
max 0, sum 5
Tm1 → TmY9q⊥ → Dm3q → TmY9q⊥
